# Evaluating the efficacy of different antibiotics against *Neisseria gonorrhoeae*: a pharmacokinetic/pharmacodynamic analysis using Monte Carlo simulation

**DOI:** 10.1186/s12879-023-08938-x

**Published:** 2024-01-18

**Authors:** Jiaojiao Zhong, Wenjing Le, Xuechun Li, Xiaohong Su

**Affiliations:** 1 Hospital for Skin Diseases, Institute of Dermatology, Chinese Academy of Medical Sciences &Peking Union Medical College, Nanjing, China; 2 Department of Dermatology, The Affiliated Hospital of Nanjing University of Chinese Medicine, Jiangsu Province Hospital of Chinese Medicine, Nanjing, China

**Keywords:** *Neisseria gonorrhoeae*, Antibiotics, Monte Carlo simulation, Pharmacokinetics/pharmacodynamics

## Abstract

**Background:**

With the widespread use of antibiotics, antimicrobial resistance in *Neisseria gonorrhoeae* is worsening. The objective of this study was to evaluate the efficacy changes of seven antibiotics in the treatment of *N. gonorrhoeae* by using Monte Carlo simulation combined with pharmacokinetics/pharmacodynamics/ (PK/PD).

**Methods:**

The minimum inhibitory concentration (MIC) of antibiotics against clinical isolates from 2013 to 2020 in Nanjing, China, was determined by agar dilution method. The probability of target attainment (PTA) was estimated at each MIC value and the cumulative fraction of response (CFR) was calculated to evaluate the efficacy of these regimens.

**Results:**

All dosage regimens of seven antibiotics achieved PTAs ≥ 90% for MIC ≤ 0.06 µg/ml. But when the MIC was increased to 1 µg/ml, PTAs at each MIC value exceeded 90% only for ceftriaxone 1,000 mg and 2,000 mg, zoliflodacin 2,000 mg and 3,000 mg. Among them, the CFR values of each dosing regimen against *N. gonorrhoeae* only for ceftriaxone, cefixime and zoliflodacin were ≥ 90% in Nanjing from 2013 to 2020.

**Conclusions:**

Cephalosporins are still the first-line drugs in the treatment of gonorrhea. However, the elevated MIC values of cephalosporins can lead to decline in clinical efficacy of the conventional dose regimens, and increasing the dose of ceftriaxone to 1,000 mg-2,000 mg may improve the efficacy. In addition, zoliflodacin is possible to be a potential therapeutic agent in the future.

## Introduction

Gonorrhea, a prevalent sexually transmitted disease, poses a significant global public health challenge. The World Health Organization(WHO)estimates that 82 million new cases of gonorrhea occurred globally in 2020 [1]. At present, *N. gonorrhoeae* is highly resistant to penicillin, ciprofloxacin, tetracycline and azithromycin, although the resistance rates may vary across different regions. In recent years, the emergence of drug-resistant strains to ceftriaxone and cefixime has been observed in multiple countries, but the first-line drug for the treatment of gonorrhea in most countries is still the third generation cephalosporins [2–5]. Recognizing the urgency, WHO listed *N. gonorrhoeae* as a priority pathogen for research and development of new antibiotics in 2017 [6]. The new antibiotics zoliflodacin and gepotidacin have shown good antibacterial ability against *N. gonorrhoeae in vitro* [7, 8], but their clinical efficacy still needs to be evaluated.

Monte Carlo simulation is a computer simulation method, and the basic idea is to build a probabilistic model, to calculate the parameters by random sampling, and finally find the estimated value of the problem [9]. For clinical antibiotics, insufficient dose may lead to treatment failure, and excessive dose may increase the pressure on liver and kidney metabolism of patients. Combining the distribution of minimum inhibitory concentration (MIC), pharmacokinetic-pharmacodynamic(PK-PD) parameters and PK-PD targets of different drugs, can calculate the probability of target attainment (PTA) and cumulative fraction of response (CFR) for different antibiotics against *N. gonorrhoeae* to evaluate the dosing regimens in antibiotic development and application [10].

In this study, we aimed to predict the efficacy of seven antibiotics (ceftriaxone, cefixime, azithromycin, penicillin, tetracycline, ciprofloxacin and zoliflodacin) in the treatment of *N. gonorrhoeae* infection in Nanjing, China based on a PK/PD model using Monte Carlo simulation analysis .

## Method

### Microorganisms and susceptibility testing

A total of 1,417 strains of *N. gonorrhoeae* were isolated from male urethritis patients in the STD clinic, Institute of Dermatology, Chinese Academy of Medical Sciences from January 2013 to December 2020. According to the Clinical and Laboratory Standards Institute(CLSI) guidelines, the MICs of ceftriaxone, cefixime, azithromycin, penicillin, tetracycline, ciprofloxacin and zoliflodacin were determined by agar dilution method (ATCC 49226 and WHO reference strains F, G, L, O and P as quality control strains in each batch of tests).

### Pharmacokinetics and pharmacodynamics indices

All pharmacokinetic parameters of all antibiotics were obtained from the published pharmacokinetic literatures (Table 1). For ciprofloxacin, tetracycline, azithromycin and zoliflodacin, fAUC_0 − 24 h_ /MIC was used as PK/PD index, and the calculation formula was as follows:

**Table 1:** Summary of PK parameters and PK/PD target level incorporated in the Monte Carlo simulation analysis

Antibiotic	Single Dose Regimen	PK/PD target level	Fauc0-t(mg.h/L)		CL(L/h)	Vd(L)	Fu	t1/2(h)	Reference
Ceftriaxone	250 mg, 500 mg, 1000 mg,im 2000 mg iv	ft > MIC>60%				14.7 ± 4.9	0.05	8.45	[21]
Cefixime	400 mg po	ft > MIC>60%				19.0 ± 3.0	0.35	3.4	[21]
Azithromycin	1000 mg, 2000 mg,po	fAuc0–24 h/MIC ≥ 59.5			144 ± 39.5				[30]
Penicillin	4,800,000U im	ft > MIC>50%				0.24	0.4–0.55	0.7–0.75	[31]
Tetracycline	500 mg po	fAuc 0–24 h/MIC ≥ 25	26.91 ± 6.01						[32]
Ciprofloxacin	500 mg po	fAuc 0–24 h/MIC ≥ 125	10.7 ± 2.6						[33]
Zoliflodacin	2000 mg, 3000 mg po	fauc0-24 h/MIC ≥ 66			16.4 ± 1,2				[7]

PK/PD: pharmacokinetics/pharmacodynamics; fauc_0 − 24 h_: free-drug 24 h area under the plasma concentration–time curve ; CL: total body clearance; Vd: volume of distribution; Fu: fraction unbound; t1/2: half-time

$$ fAU{C_{0 - 24h}}/MIC = {f_{dose24h}}/\left( {CL \times MIC} \right)$$

fAUC_0 − 24 h_ is the 24-hour free drug area under the plasma concentration-time curve, dose is the drug dose, f is the fraction of free drug in the plasma, CL is the total body clearance, and MIC is the minimum inhibitory concentration.

For cefixime, ceftriaxone and penicillin, %fT_> MIC_ was used as the PK/PD index, the calculation formula is as follows:

$$ \% f{T_{ > MIC}} = ln\left( {\frac{{Dose \times fu}}{{Vd \times MIC}}} \right) \times \frac{{Vd}}{{CL}} \times \frac{{100}}{{DI}}$$

Fu is the fraction of unbound drug, Vd is the volume of distribution at steady state, and DI is dosing interval.

### Monte Carlo simulation

Monte Carlo simulations were performed for 5,000 trials using Crystal Ball software (version 11.1) based on pharmacokinetic parameters and different distributions of MICs. Pharmacokinetic parameters were assumed to follow a lognormal distribution and MICs were discretely distributed, and the PK/PD indices were calculated for each simulation. The probability of target attainment (PTA) at each MIC value of each regimen was obtained according to the PK/PD index, and the PTA represents the likelihood that an antibacterial regimen meets or exceeds a specific MIC target. The cumulative fraction of response (CFR) represents the probability that a specific drug dose achieves the desired target for the entire microbiota, and a regimen with CFR greater than 90% were considered optimal and is calculated as follows:

$$CFR = \sum\nolimits_{i = 1}^n {PTAi \times Fi} $$

PTAi represents the probability of antibiotic effectiveness corresponding to each MIC value, and Fi represents the proportion of strains with this MIC.

### The results of susceptibility testing

Table 2 shows the MIC_50_, MIC_90_, MIC range and drug resistance rate of *N. gonorrhoeae* isolated in Nanjing from 2013 to 2020. The resistance rate of *N. gonorrhoeae* to ceftriaxone and cefixime in Nanjing was zero in 2013–2016, indicating good antimicrobial activity but both showed a resistance rate of 2.1% from 2017 to 2020. The resistance rates of azithromycin, penicillin, tetracycline, and ciprofloxacin in Nanjing were 17%, 78.5%, 95.8% and 100% in 2017–2020, with resistance rates of 10.2%, 83%, 92% and 100% in 2017–2020. The MIC value of zoliflodacin was 0.002 ~ 0.25 µg/ml, which is lower than the MIC value of other commonly used antibiotics against *N. gonorrhoeae*. However, the MIC breakpoint for zoliflodacin remains unknown, and resistance rates could not be obtained.

**Table 2:** MIC distributions for antimicrobials against *Neisseria gonorrhoeae* in Nanjing from 2013 to 2020

Antibiotic	Area&Year	MIC_50_(µg/ml)	MIC_90_(µg/ml)	MIC range(µg/ml)	Resistance rate (%)
Ceftriaxone	Nanjing 2013–2016	0.03	0.125	0.002–0.25	0
	Nanjing 2017–2020	0.06	0.125	0.002-1	2.1
Cefixime	Nanjing 2013–2016	0.03	0.125	0.002-0.5	0
	Nanjing 2017–2020	0.06	0.25	0.004->4	2.1
Azithromycin	Nanjing 2013–2016	0.5	16	0.015–8192	17
	Nanjing 2017–2020	0.25	2	0.015-512	10.2
Penicillin	Nanjing 2013–2016	4	>8	0.125->8	78.5
	Nanjing 2017–2020	4	>8	0.125->8	83
Tetracycline	Nanjing 2013–2016	4	>32	0.125->32	95.8
	Nanjing 2017–2020	2	>32	0.25->32	92
Ciprofloxacin	Nanjing 2013–2016	>8	>8	1->8	100
	Nanjing 2017–2020	>8	>8	1->8	100
Zoliflodacin	Nanjing 2013–2016	0.06	0.06	0.002–0.125	NA
	Nanjing 2017–2020	0.06	0.125	0.002–0.25	NA

### Probability of target attainment

Table 3 shows the PTA of each antibiotic dosing regimen at different MIC values. All antimicrobial dosage regimens achieved PTAs ≥ 90% for MIC ≤ 0.06 µg/ml. Conventional dose regimens of ceftriaxone 250 mg and 500 mg failed to reach PTA ≥ 90% against a MIC of 0.5 µg/ml and 1 µg/ml, while higher dose of ceftriaxone for 1,000 mg and 2,000 mg can achieved PTA ≥ 99.09% and 100% against the MIC ≤ 1 µg/ml. The PTA was ≥ 99.73% and 100% for cefixime at doses of 400 mg and 800 mg when the MIC was ≤ 0.25 µg/ml and 0.5 µg/ml, respectively. Similarly, azithromycin at doses of 1,000 mg and 2,000 mg achieved PTA ≥ 100% and 99.18% when the MIC was ≤ 0.06 µg/ml and 0.125 µg/ml. Penicillin at a dose of 4.8 million units, tetracycline 500 mg and ciprofloxacin 500 mg resulted in a PTA ≥ 100%, 99.3% and 91.32% when the MIC was ≤ 0.25 µg/ml, 0.5 µg/ml and 0.06 µg/ml, respectively. In contrast, zoliflodacin 2,000 mg and 3,000 mg can achieve PTA ≥ 99.12% and 100% at MIC ≤ 2 µg/ml. The MIC values of all antibiotics at the PTA > 90% critical point were equal to or close to the susceptibility breakpoints. Therefore, it can be inferred that the drug susceptibility point of zoliflodacin may be around 2 µg/ml based on the PK-PD parameters.

**Table 3:** Probability of target attainment (PTA) at each MIC in different antibiotics dose regimen

Antibiotic Single Dose Regimen	PTA (%) at each MIC(µg/ml)										
	0.03	0.06	0.125	0.25	0.5	1	2	4	8	16	
Ceftriaxone 250 mg im	100	100	100	99.32	62.15	3.08	0	0	0	0	
Ceftriaxone 500 mg im	100	100	100	100	99.17	62.65	2.85	0	0	0	
Ceftriaxone 1,000 mg im	100	100	100	100	100	99.09	61.96	3.08	0	0	
Ceftriaxone 2,000 mg iv	100	100	100	100	100	100	99.45	61.58	3.69	0	
Cefixime 400 mg po	100	100	100	99.73	6.98	0	0	0	0	0	
Cefixime 800 mg po	100	100	100	100	100	5.49	0	0	0	0	
Azithromycin 1,000 mg po	100	100	45.37	0	0	0	0	0	0	0	
Azithromycin 2,000 mg po	100	100	99.18	44.72	0	0	0	0	0	0	
Penicillin 4,800,000U im	100	100	100	100	74.95	0	0	0	0	0	
Tetracycline 500 mg po	100	100	100	100	99.3	62.99	0	0	0	0	
Ciprofloxacin 500 mg po	100	91.32	4.71	0	0	0	0	0	0	0	
zoliflodacin 2,000 mg po	100	100	100	100	100	100	99.12	0	0	0	
zoliflodacin 3,000 mg po	100	100	100	100	100	100	100	39.64	0	0	

### Cumulative fraction of response

Table 4 shows the CFR values of each antibiotic regimen against gonococci in Nanjing, China based on Monte Carlo simulation. Among them, the CFR values of each dosing regimen of ceftriaxone and cefixime were ≥ 93.79%. However, regardless of the regimen, the CFRs of cefixime and ceftriaxone in Nanjing decreased from 2017 to 2020 compared with those from 2013 to 2016, but an increase in dosage could result in a corresponding increase in CFR values. In contrast, the CFR for each dosing regimen of azithromycin, penicillin, tetracycline, and ciprofloxacin was ≤ 55.33%, 5.4%, 13.61% and 0% from 2013 to 2020 in Nanjing, respectively. In addition, the dosing regimen of zoliflodacin 2,000 mg and 3,000 mg in Nanjing from 2013 to 2020 both has a CFR value of 100%.

**Table 4:** Cumulative fraction of response (CFR) for achieving PK/PD index with different antimicrobials against *Neisseria gonorrhoeae* in Nanjing, China from 2013 to 2020

Antibiotic	Single Dose Regimen	CFR(%)		
		2013–2016	2017–2020	
Ceftriaxone	250 mg im	99.75	96.45	
	500 mg im	99.99	97.07	
	1000 mg im	100	98.19	
	2000 mg iv	100	98.81	
Cefixime	400 mg po	98.35	93.79	
	800 mg po	100	97.93	
Azithromycin	1000 mg po	24.88	19.52	
	2000 mg po	43.51	55.33	
Penicillin	4,800,000U im	5.4	3.35	
Tetracycline	500 mg po	10.03	13.61	
Ciprofloxacin	500 mg po	0	0	
Zoliflodacin	2000 mg po	100	100	
	3000 mg po	100	100	

## Discussion

Gonorrhea is widespread throughout the world, and the susceptibility of *N. gonorrhoeae* to antibiotics varies by the region and time. The susceptibility of *N. gonorrhoeae* is analyzed by the MIC values combined with the susceptibility breakpoints, while the susceptibility breakpoints need to be determined in combination with in vitro microbiological data, PK/PD parameter, as well as clinical outcomes [11]. The aim of this study was to calculate the PTAs of several antibiotics at different MIC values of *N. gonorrhoeae* by Monte Carlo simulation combined with PK/PD, and to analyze the different efficacy of various antibacterial regimens, so as to guide the application of antibiotics in *N. gonorrhoeae* .

The data of resistance rates to penicillin (78.5%–83%), tetracycline (92%-95.8%) and ciprofloxacin (100%) in Nanjing from 2013 to 2020 indicated that antimicrobial resistance of *N. gonorrhoeae* is serious in Nanjing, China, which may be related to the abuse of antibiotics in China [12]. This result is consistent with the results of antibiotic susceptibility testing of *N. gonorrhoeae* in other regions of China [13–15]. Furthermore, when the MIC value exceeded 0.5 µg/ml, 1 µg/ml, 0.125 µg/ml, respectively, the corresponding PTA of penicillin 480,000U, tetracycline 500 mg, ciprofloxacin 500 mg was less than 90%, indicating that theses antibiotics dosage regimen may have poor efficacy when the MIC values of *N. gonorrhoeae* is elevated. It is well known that penicillin, tetracycline, and ciprofloxacin are no longer recommended for the treatment of gonorrhea [2]. Dual therapy with ceftriaxone and azithromycin has been previously recommended in the treatment of gonorrhea [16]. According to the data in this study, the azithromycin resistance rate in Nanjing area reached 10.2–17%, but the PTA for azithromycin 1,000 mg and 2,000 mg dose regimens was ≤ 90% when the MIC values were ≥ 0.125 µg/m and 0.25 µg/ml, respectively. The United States Centers for Disease Control and Prevention (CDC) has removed azithromycin from treatment recommendations in December 2020 due to increasing resistance rate [17].

Third-generation of cephalosporins remain the first-line drugs recommended by the WHO for the treatment of gonorrhea [18]. In Nanjing, the resistance rate of ceftriaxone or cefixime was zero in 2013–2016, but resistant strains appeared in 2017–2020, with a resistance rate of 2.1%. In other areas of China the drug resistance rate of ceftriaxone in Hangzhou was 3% during 2015–2017 [19], and 9.87% in South China during 2016–2020 [20]. With the widespread use of drugs, the MIC value of ceftriaxone keeps rising. The MIC value of ceftriaxone was up to 1 µg/ml in Nanjing from 2017 to 2020. Monte Carlo simulation combined with PK/PD analysis was conducted to adjust the recommended first-line drug dose according to changes in the distribution of *N. gonorrhoeae* MIC values [21]. Increasing the dose of ceftriaxone may be an effective solution to the problem of elevated MIC values. When resistant strains with MIC values over 1 µg/ml of ceftriaxone appear, the PTA of ceftriaxone 250 mg and 500 mg is less than 3.08% and 62.15% respectively, which may lead to treatment failure. Furthermore, when the dose of ceftriaxone was increased to 1,000 mg and 2,000 mg, the corresponding PTA can be elevated to 99.09% and 100% at the MIC of 1 µg/ml, so it is recommended to increase the clinical dose of ceftriaxone to improve the efficacy. Some countries have recommended higher doses of ceftriaxone as monotherapy for gonorrhea. For example: the United States recommended ceftriaxone 500 mg single intramuscular injection [3], while both the United Kingdom and China recommended ceftriaxone 1,000 mg single intramuscular injection [22, 23]. Ceftriaxone 2,000 mg is still well tolerated in patients with community-acquired pneumonia [24]. In 2015, the FC428 strain of *N. gonorrhoeae* resistant to ceftriaxone was first isolated from a male urethritis patient in Japan, with an MIC of 0.5 µg/ml ; since then, FC428-like isolates have been identified in many countries (including China) [25]. In 2020, ceftriaxone resistant FC428 strains (MIC = 1 µg/ml) were found in Hangzhou, China, which is failed to be treated with conventional doses of cephalosporins. Among them, three patients had negative culture results after intravenous injection of 2,000 mg ceftriaxone for 1 or 2 days [26]. However, higher dose can result in an increased incidence of gastrointestinal adverse effects [27]. Cefixime is not recommended as the first-line treatment for gonorrhea in Chinese guidelines, and the PTA of cefixime 400 mg and 8008 mg for MIC ≥ 1 µg/ml is less than 5.49%. However, the CFR of cefixime 400 mg in Nanjing decreased from 98.35% in 2013–2016 to 93.79% in 2017–2020. Increasing the cefixime dose from 400 mg to 800 mg could improve the CFR, but higher dose can result in an increased incidence of gastrointestinal adverse effects [27]. Therefore, as the widespread of gonococcal drug resistance, increasing the dose of cephalosporins for gonorrhea can be considered in the future, but the adverse reactions caused by increasing the dose and the induction of drug resistance need to be considered.

Zoliflodacin is a new class of spiropyrimidinetriones antibiotic, which targets the GyrB subunit in DNA gyrase to stabilize the cleavage covalent complex between DNA gyrase and double-strand broken DNA, preventing the formation of circular DNA [28]. In this study, the PTA of zoliflodacin 2000 mg and 3000 mg can exceed 99.12% and 100% at MIC ≤ 2 µg/ml, and the CFR of *N. gonorrhoeae* in Nanjing area from 2013 to 2020 was 100% for oral administration of 2,000 mg and 3,000 mg zoliflodacin. The results of phase 2 clinical trial showed that the cure rates of a single oral dose of 2,000 mg or 3,000 mg zoliflodacin for urogenital gonorrhea were 98% and 100%, respectively, 100% for rectal gonorrhea, 67% and 78% for pharyngeal gonorrhea [29]. Combined with Monte Carlo simulations, appropriate doses for phase 2 and 3 clinical trials can be predicted based on PK/PD parameters obtained from phase 1 clinical trials. A larger scale of phase 3 trial is undergoing to evaluate the safety and efficacy of a 3,000 mg oral dose of zoliflodacin compared to a combination of a single intra-muscular 500 mg dose of ceftriaxone and a single 1 g oral dose of azithromycin for the treatment of uncomplicated gonorrhea. In addition, based on the PK-PD targets under different MIC values, it is helpful to determine the susceptibility breakpoint of antibiotics [9]. The MIC value corresponding to PTA > 90% can serve as a reference for establishing the antibiotic susceptibility breakpoint, but it should be complemented with additional data from clinical studies. When the PTA of zoliflodacin is ≥ 90%, the MIC value for the 2,000 mg and 3,000 mg dose is ≤ 2 µg/ml. It can be preliminarily inferred that the drug susceptibility breakpoint of zoliflodacin is around 2 µg/ml, but this inference still requires a larger amount of clinical data to support and validate.

There are several limitations in this study. First, the PK/PD parameters in this study come from published clinical studies, and these data may have some heterogeneity due to different study populations, which may lead to the occurrence of bias. Secondly, the MIC data in this study were all from Nanjing area, and there were differences in the susceptibility of *N. gonorrhoeae* to antibiotics in different regions, so this study can only represent the prediction of the drug susceptibility of *N. gonorrhoeae* in Nanjing. Thirdly, some people have other diseases, such as liver and kidney insufficiency or hypoproteinemia, which will affect the metabolism of antibiotics and lead to inaccurate prediction results. Fourth, this study did not predict the effect of antibiotic combination therapy. For the effect of new type antibacterial drugs, this study only predicts the effect of zoliflodacin, and there is a lack of data for gepotidacin and solithromycin, which are also in clinical trials stage. Therefore, more large-sample studies are needed to determine the clinical value of these findings and recommendations.

## Conclusion

At present, cephalosporins are still the first-line drugs for the treatment of gonorrhea. However, the increased MIC value of *N. gonorrhoeae* for cephalosporins may lead to decline in clinical efficacy of the conventional dose regimens, and the Monte Carlo simulation results show that increasing the dose of ceftriaxone to 1000 mg-2000 mg may improve the efficacy. In addition, zoliflodacin is possible to be potential treatment for gonorrhea in the future, but more clinical results are still needed to verify this conclusion.

## Data Availability

The data that support the findings of this study are available from the corresponding author, upon request.
